# Cranial and spinal nerve enhancement in *SURF1*-associated Leigh syndrome

**DOI:** 10.1007/s00247-024-06005-4

**Published:** 2024-07-27

**Authors:** Mhairi Dupré, Richard Warne, Peter Shipman, Maina Kava, Twinkle Ghia, Lily Loughman, Rahul Lakshmanan

**Affiliations:** 1 Medical Imaging Department, Perth Children’s Hospital, Perth, 6009 Australia; 2 Department of Neurology, Perth Children’s Hospital, Perth, 6009 Australia; 3 Department of Metabolic Medicine, Perth Children’s Hospital, Perth, 6009 Australia; 4 School of Paediatrics and Child Health, UWA Medical School, University of Western Australia, Perth, 6009 Australia; 5 Genetic Health Western Australia, King Edward Memorial Hospital, Perth, 6008 Australia; 6 UWA Medical School, Centre for Neuromuscular and Neurological Disorders (Perron Institute), Nedlands, 6009 Australia

**Keywords:** Leigh syndrome, Magnetic resonance imaging, Mitochondrial disorders, Nerve enhancement, Pediatric, *SURF1*

## Abstract

A 23-month-old boy with poor growth, developmental delay, and hypotonia presented with acute onset of ataxia and fatigue. Magnetic resonance imaging (MRI) of the brain and spinal cord was performed as part of diagnostic work-up. MRI showed bilateral symmetrical lesions in basal ganglia, midbrain, and brainstem consistent with Leigh syndrome. Signal abnormalities were also present within the cervical cord, with enhancement of multiple cranial, spinal, and cauda equina nerve roots. Genetic testing confirmed compound heterozygosity for two pathogenic variants in *SURF1* implicated in Leigh syndrome. Whilst nerve root enhancement has been described in other mitochondrial disorders, we believe this is the first published case of both cranial and spinal nerve root enhancement in Leigh syndrome.

**Graphical Abstract:**

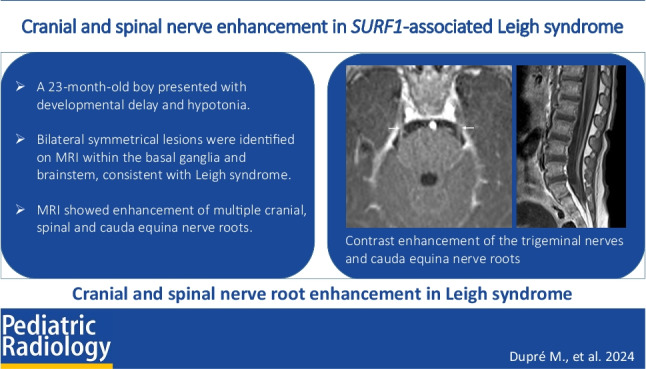

## Introduction

Mitochondria are complex intracellular organelles essential for various functions including energy production, fatty acid oxidation, and programmed cell death. Inherited mitochondrial disorders are a heterogeneous group of diseases due to variants in both mitochondrial and nuclear DNA. Many inherited mitochondrial disorders are primarily progressive with high morbidity and mortality, the severest forms being lethal in utero or infancy. The onset of clinical symptoms may be triggered by physiological stress such as infection or dehydration [1].

Leigh syndrome, also known as subacute necrotizing encephalomyelopathy, is a neurodegenerative disorder encompassing disruptions to the mitochondrial electron transport chain, involved in ATP production, and as such commonly affects locations within the central nervous system with the highest energy requirements [2]. Children commonly present before the age of 2 years with psychomotor delay or regression, hypotonia, and signs of basal ganglia or brainstem dysfunction, which may manifest as swallowing and respiratory difficulties, ataxia, nystagmus, or ophthalmoplegia; death is usually before 10 years of age [2]. Diagnosis involves magnetic resonance imaging (MRI) of the neuroaxis, identifying symmetrical signal abnormalities within the basal ganglia, thalami, and brainstem. Pathologically this is correlated with spongiform degeneration, demyelination, gliosis, and neuronal loss. Serum or cerebrospinal fluid (CSF) may display elevation of lactate [1].

Surfeit locus protein 1 (*SURF1*) encodes an assembly factor maintaining cytochrome c oxidase stability. Cytochrome c oxidase deficiency due to loss of function variants in *SURF1* has been implicated in both Leigh syndrome (the clinical manifestation of mitochondrial complex IV deficiency, nuclear type 1; OMIM 220110) and Charcot-Marie-Tooth disease (type 4 K; OMIM 616684). More than 100 different variants are recorded in *SURF1*-associated Leigh syndrome, and although neuroimaging features may vary, *SURF1*-related phenotypes often spare the putamina, favouring greater involvement of the brainstem. Leukoencephalopathy and cerebellar atrophy are common features, with or without dentate nucleus involvement [1].

Despite these typical MRI findings, demonstrably Leigh syndrome can involve any level of the neuroaxis, with atypical presentations. Whilst there are a few reports describing inherited mitochondrial disorders with acute signal abnormalities in spinal [3–5] and cranial [3, 4, 6] nerves, as far as we are aware, this is the first published case of both cranial and spinal nerve involvement in Leigh syndrome.

## Case report

A 23-month-old non-dysmorphic boy was under investigation for developmental delay, hypotonia, and feeding difficulties. Born at term via emergency Caesarean section for fetal distress, to non-consanguineous parents, there had been no perinatal issues. Following sudden onset of ataxia, fatigue, and myoclonus, the child was admitted to hospital and found to be alert with no visual or hearing deficits, but had gross and fine motor delay, central and peripheral hypotonia, and involuntary truncal/peripheral choreiform movements. Despite absent lower limb reflexes, the patient had normal nerve conduction studies.

CSF showed elevated lactate of 3.1 mmol/L (normal range < 2.0 mmol/L) and elevated protein of 0.79 g/L (normal range 0.10–0.35 g/L), but no micro-organisms were cultured. Inflammatory markers were mildly elevated with C-reactive protein 16 mg/L, neutrophils 9.7 x10^9/L. Family history was non-contributory, and previous creatinine kinase measurements, electroencephalogram, and hearing test were normal.

**Fig. 1:**
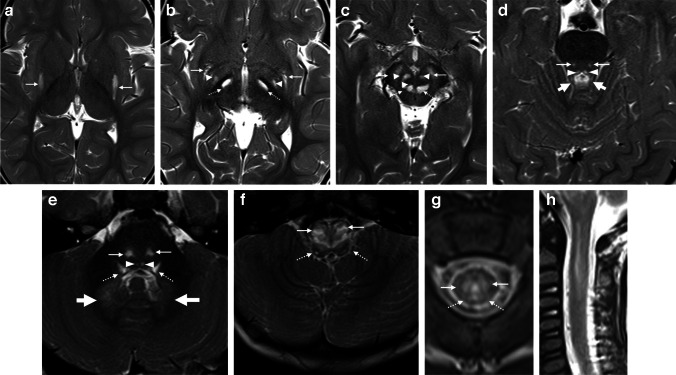
T2-weighted magnetic resonance images through the brain demonstrate features of Leigh syndrome in a 23-month-old boy with confirmed *SURF1* variants. **a**–**c **Axial images demonstrate T2 hyperintensity in the bilateral posterior putamina (*arrows* in **a**), globi pallidi interna (*arrowheads* in **b**), subthalamic nuclei (*broken arrows* in **b**), substantia nigra (*arrows* in **c**), red nuclei (*arrowheads* in **c**), and periaqueductal grey matter (*broken arrows* in **c**). **d**–**f** Axial images at the level of superior cerebellar peduncles (**d**), middle cerebellar peduncles (**e**), and medulla (**f**) demonstrate hyperintensity within the central tegmental tracts (*arrows* in **d** and **e**), medial longitudinal fasciculi (*arrowheads* in **d** and **e**), trigeminal sensory nuclei (*broken arrows* in **e** and **f**), cerebellar dentate nuclei (*thick arrows* in **e**), and inferior olivary nuclei (*arrows* in **f**). **g**, **h** Axial (**g**) and sagittal (**h**) images demonstrate spinal cord involvement with increased signal in the central cord grey matter (*arrows*) and dorsal columns (*broken arrows*)

An MRI of the brain showed fairly symmetrical but widespread grey matter involvement, with increased T2/fluid attenuated inversion recovery (FLAIR) signal within the posterior putamina, subthalamic nuclei, cerebral peduncles, midbrain (periaqueductal grey matter and inferior colliculus), pons (central tegmental tracts and medial longitudinal fasciculus), superior cerebellar peduncles, dentate hilus, and medulla (with focal expansion of the inferior olivary nuclei) (Fig. 1).

**Fig. 2:**
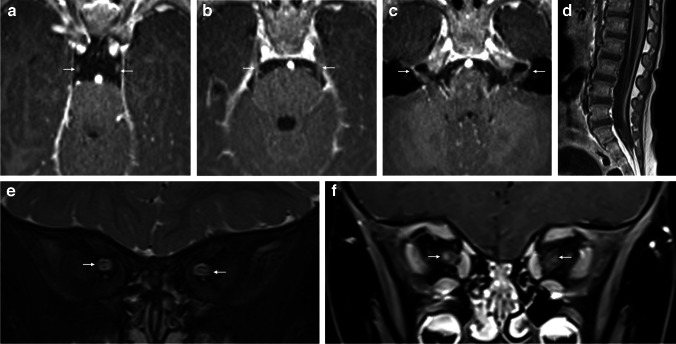
Cranial nerve involvement in a 23-month-old boy with Leigh syndrome and confirmed *SURF1* variants. **a**–**c** Axial T1 post-gadolinium images show enhancement of the oculomotor nerves (*arrows* in **a**), trigeminal nerves (*arrows* in **b**), and facial nerves (*arrows* in **c**). **d** Sagittal post-gadolinium image demonstrates diffuse thickening and enhancement of the cauda equina nerve roots. **e** Coronal T2 fat saturated image shows small T2 hyperintense optic nerves (*arrows*). **f** Coronal T1 post-gadolinium Dixon image shows associated optic nerve enhancement (*arrows*)

Punctate foci of restricted diffusion were visible in the pallidi, subthalamic nuclei, cerebral peduncles, midbrain (red nuclei), dorsal pons (central tegmental tracts), superior cerebellar peduncles, dentate nuclei, and medulla anteriorly (inferior olivary nuclei) (Fig. 2). Heterogeneous enhancement was only visible within the medulla. The white matter was spared. MR spectroscopy showed elevated lactate peaks.

Abnormalities in multiple cranial nerves were visible, most markedly the thinned and atrophic optic nerves, which displayed high T2/FLAIR signal and bilateral retrobulbar and pre-chiasmatic post-contrast enhancement. There was also pathological enhancement of the cisternal oculomotor, cisternal (and within Meckel’s cave) trigeminal nerves, and bilateral cisternal and labyrinthine facial nerves.

Spinal cord imaging showed central cord grey matter and dorsal column cervical cord increased T2 signal, extending between the levels of cervical (C) vertebra 2–7, with subtle central cord enhancement at the C2–C3 level. There were thickened enhancing cauda equina nerve roots as well as enhancing cervical and thoracic spinal nerves.

Massively parallel sequencing of a custom neuromuscular gene panel identified two heterozygous variants in *SURF1*, c.312_321delinsAT p.(Leu105*) and c.574C > T p.(Arg192Trp), predicted and demonstrated to result in loss of function respectively. Both variants were classified as pathogenic according to American College of Medical Genetics guidelines and presumed in trans, though no parental analysis has yet been performed.

The patient was discharged but presented again a few weeks later with metabolic decompensation in the context of rhinovirus infection, and started on a ketogenic diet for Leigh syndrome, with regular serum lactate and urinary ketone testing. Following improvement of acute symptomology, the patient was discharged on a modified ketogenic diet, with urinary ketone monitoring. Alerts were entered into the electronic record to avoid IV dextrose in the event of future acute illness. Neurology and clinical genetics are to be followed-up in outpatient clinics.

## Discussion

We believe this is the first published case of both cranial and spinal nerve root enhancement in Leigh syndrome, and only the second case of concomitant cranial and spinal nerve enhancement in a  child with a mitochondrial disorder. The first case was reported by Horst et al. on a background of polymerase-gamma (*POLG*) variant without a Leigh syndrome presentation [4].

Cranial and spinal nerve root pathological enhancement is rare in children, usually as a result of an inflammatory, traumatic, or malignant process. Whilst cranial nerve enhancement has been documented in other childhood metabolic disorders including metachromatic leukodystrophy and Krabbe’s disease [4], it is a very rare feature of mitochondrial disorders, with only five cases documented in the literature, the majority (three) being adults. Of these five, two case studies were of individuals with *POLG* variants [3, 4], and two were of patients with mitochondrial neuro-gastro-intestinal encephalopathy syndrome (*TYMP*; OMIM 603041) [6, 7], the latter cases displaying only cranial nerve and no spinal nerve involvement. A single case has described enhancing spinal nerve roots in a patient with *SURF1* variants [5].

The reason for nerve enhancement is yet unknown; active demyelination, perivascular inflammation, or cytotoxic metabolite accumulation have all been postulated [8]. Whilst peripheral neuropathy in adults with mitochondrial disease has been well described [8], it is not common in children, particularly rare in those with Leigh syndrome or *POLG*-related disorders.

Many children having neuroimaging for metabolic disorders have MRIs without intravenous contrast, leading to under reporting of nerve root enhancement. Given that prior to this current publication, concomitant cranial and spinal nerve enhancement in inherited mitochondrial disorders had only been identified in *POLG*-related disorders, this has previously been postulated to be a differentiating factor for these variants [3]. However, in this article we present cranial nerve enhancement in Leigh syndrome, confirming that it is not a unique feature of *POLG*-related disorders. We advise considering post-contrast sequences of the neuroaxis as part of the diagnostic work-up in all children where a neurometabolic disorder is suspected.
